# Supplementary material for: Immunolocalization of Nesfatin-1 in the Gastrointestinal Tract of the Common Bottlenose Dolphin Tursiops truncatus
Source: Animals (Basel). 2022 Aug 22;12(16):2148. doi: 10.3390/ani12162148 (PMC9405072; doi:10.3390/ani12162148)
Supplement: Supplementary file 1 [file animals-12-02148-s001.zip › animals-1685731-supplementary.pdf]

Immunolocalization of Nesfatin-1 in the gastrointestinal tract of the common bottlenose dolphin *Tursiops truncatus*

Elena De Felice[†1*], Claudia Gatta[†2], Daniela Giaquinto[2], Federica Fioretto[1], Lucianna Maruccio [2], Danila d'Angelo[2], Paola Scocco[1], Paolo de Girolamo[2] and Livia D'Angelo[2]

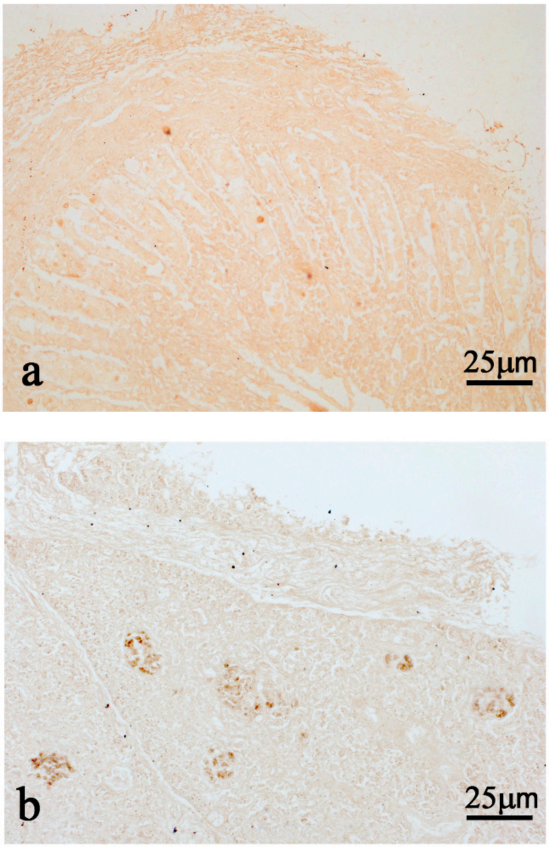

Figure S1. Immunohistochemistry negative and positive controls of anti-Nesf-1 in *Tursiops truncatus*. Negative control of intestine **(a)**. Positive control of pancreas, showing Nesf-1 positive immunoreactivity in pancreatic islet **(b)**.

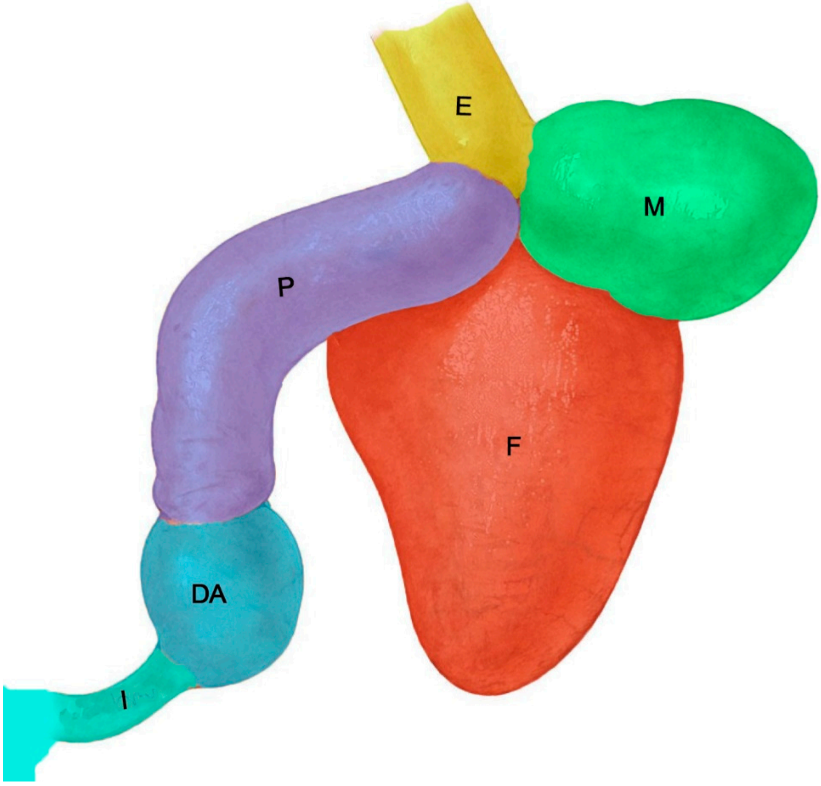

Figure S2. Schematic drawing of the gastrointestinal tract of the *Tursiops truncatus*: (E) esophagus; (F) forestomach or first chamber; (M) main stomach or second chamber; (P) pyloric stomach or third chamber, (DA) duodenal ampulla and (I) intestine.
